# Multifunctional GaFeO_3_ Obtained via Mechanochemical Activation Followed by Calcination of Equimolar Nano-System Ga_2_O_3_–Fe_2_O_3_

**DOI:** 10.3390/nano11010057

**Published:** 2020-12-29

**Authors:** Lucian Diamandescu, Felicia Tolea, Marcel Feder, Florin Vasiliu, Ionel Mercioniu, Monica Enculescu, Traian Popescu, Bogdan Popescu

**Affiliations:** National Institute of Materials Physics, Atomistilor 405A, 077125 Magurele, Romania; diamand@infim.ro (L.D.); mfeder48@gmail.com (M.F.); fvasiliu@infim.ro (F.V.); imercioniu@infim.ro (I.M.); mdatcu@infim.ro (M.E.); tr.popescu@googlemail.com (T.P.); bogdan.popescu@infim.ro (B.P.)

**Keywords:** GaFeO_3_, high-energy ball milling, Mössbauer spectroscopy, TEM, SAED, UV-vis optical and magnetic properties

## Abstract

The equimolar oxide mixture β-Ga_2_O_3_—α-Fe_2_O_3_ was subjected to high-energy ball milling (HEBM) with the aim to obtain the nanoscaled GaFeO_3_ ortho-ferrite. X-ray diffraction, ^57^Fe Mössbauer spectroscopy, and transmission electron microscopy were used to evidence the phase structure and evolution of the equimolar nano-system β-Ga_2_O_3_—α-Fe_2_O_3_ under mechanochemical activation, either as-prepared or followed by subsequent calcination. The mechanical activation was performed for 2 h to 12 h in normal atmosphere. After 12 h of HEBM, only nanoscaled (~20 nm) gallium-doped α-Fe_2_O_3_ was obtained. The GaFeO_3_ structure was obtained as single phase, merely after calcination at 950 °C for a couple of hours, of the sample being subjected to HEBM for 12 h. This temperature is 450 °C lower than used in the conventional solid phase reaction to obtain gallium orthoferrite. The optical and magnetic properties of representative nanoscaled samples, revealing their multifunctional character, were presented.

## 1. Introduction

Gallium iron oxide, GaFeO_3_ (GFO), exhibits ferromagnetic and ferroelectric properties and has been studied for its promising applications as a multiferroic material [1]. This group of materials could have many applications, e.g., visible light water splitting [2] or a new random access memories generation. GFO crystallizes in the orthorhombic structure, space group (S.G.) number 33, Pna2_1_ (Inorganic Crystal Structure Data (ICSD)). Commonly, the GFO is prepared by a solid-state reaction between β-Ga_2_O_3_ and α-Fe_2_O_3_ at relative high temperature (~1400 °C) and long reaction time (5–20 h) [1]. Ga_2_O_3_ is an important wide band gap (*E_g_* > 3 eV) semiconducting material presenting five polymorphous *α*, *β*, *γ*, *δ*, and *ε* phases. The β-Ga_2_O_3_ with monoclinic structure (ICSD, S.G. 12, C 1 2/m 1) is thermodynamically stable and used in optoelectronic devices [3], high-temperature gas sensors [4], and heterogeneous photocatalysis [5]. α-Fe_2_O_3_ (hematite) is a versatile semiconducting material with applications from catalysis [6] and photocatalysis [7] to gas sensing [8], depending on morphology, preparation route and doping, owing to a favorable band gap energy (2.1 eV), chemical stability, natural abundance, low cost, and no toxicity. α-Fe_2_O_3_ crystallizes in the rhombohedral corundum structure (ICSD, S.G. 167, R3¯c).

Different preparation routes have been employed for obtaining GFO, such as solid-state reactions or chemical routes, which are shown to significantly influence the properties. Powders of Ga_x_Fe_2−x_O_3_ can be obtained by the conventional solid-state reaction technique [1] and a combination of reverse-micelle and sol-gel methods (RMSG) [9,10,11]. A modified Pechini method was proposed by T.C. Han et al. [12], where the obtained precursory powders were reground and sintered at 800 °C for 2 h. Epitaxial GFO thin films were prepared via sol-gel method, exhibiting a high purity degree [13,14]. GaFeO_3_ as single crystal has been prepared using the high oxygen pressure floating zone method (HPFZ) [15]. GFO nanofibers [16] with different molar ratios of Ga:Fe were synthesized by sol-gel based electrospinning. 

In the last few decades, the mechanical milling technique has been recognized as a method to obtain nanoscaled materials in which extended solid solutions or non-equilibrium phases can be formed at friendly temperatures [17,18,19,20,21]. Recent papers [22,23,24] report the synthesis of some ferrite systems directly by a high-energy ball milling technique (HEBM); e.g., starting with ZnO–Fe_2_O_3_ [22], La_2_O_3_–Fe_2_O_3_ [23], Eu_2_O_3_–Fe_2_O_3_ [24] oxide mixtures, the HEBM process leads directly to the formation of ZnFe_2_O_4_, LaFeO_3_, and EuFeO_3_, respectively. 

The primary goal of our study was to obtain GFO by only employing energetic ball milling. The first part of the paper reports on the attempt to obtain GFO by HEBM of the equimolar mixture β-Ga_2_O_3_ and α-Fe_2_O_3_; the second part presents the successful synthesis of GaFeO_3_ by HEBM followed by calcination at temperatures hundreds of degrees lower than those required by the classical process. Compared to the sol-gel preparation route, which employs similar temperatures, much larger quantities of GFO can be easily obtained by our method. The phase evolution is presented along with the peculiar characteristics and properties of the initial, intermediate and final products.

## 2. Materials and Methods 

Equimolar quantities of β-Ga_2_O_3_ (Fluka 99.99%) and α-Fe_2_O_3_ (Merck 99.5%) were homogenized in acetone (magnetic stirrer), then evaporated at 50 °C on the drying stove. The HEBM process was performed in a SPEX 8000 M device (SPEX SamplePrep LLC, Metuchen, NJ, USA) equipped with a motor working at 1435 rpm (230 V), for time periods ranging from 2 to 12 h, at room temperature (23 °C). The experiments were performed in a hardened steel vial using ½ in. and ¼ in. steel balls, at 10/1 balls to powder mass ratio. Seven 1/4 in. and three 1/2 in. balls were used together in the HEBM experiments. Subsequently, the samples were subjected to thermal treatments, in the range of 600–1000 °C in an oven made by CALORIS GROUP SA, Bucharest, Romania.

Bruker D8 Advance X-ray diffractometer (Bruker, Hamburg, Germany) with CuK_α_ radiation, λ = 1.5406 Å and Lithium fluoride (LiF) monochromator, was used to obtain the diffraction patterns of the prepared samples. The diffractograms were recorded at room temperature (23 °C) in Bragg-Brentano geometry. After a first evaluation using Bruker AXS DIFFRAC.EVA (Bruker AXS, Karlsruhe, Germany, 2000), Rietveld refinement was applied, in the hypothesis of Pseudo-Voigt profile of the lines. Transmission electron microscopy (TEM) was also employed to obtain specific information about the structure and morphology of the mixed oxide system. TEM and high resolution transmission electron microscopy (HRTEM) images were recorded on a JEOL JEM ARM 200 F electron microscope (JEOL Ltd, Tokyo, Japan), operating at an accelerating voltage of 200 kV. Samples for transmission electron microscopy (TEM) were prepared by suspending them in ethanol and transferring to a copper grid coated with an amorphous carbon support. The particles’ sizes were established from the measurement of ~100 particles for each sample. 

The Mössbauer spectra were recorded at room temperature using a WissEL-ICE Oxford Mössbauer cryomagnetic system (Wissenschaftliche Elektronik GmbH, Starnberg, Germany, and ICE Innovative cryogenic system, Oxford, UK) with ^57^Co source in Rhodium matrix, in constant acceleration mode and velocity range (−10–+10) mm/s. α-Iron foil was used to calibrate the spectrometer. 

Optical properties of the investigated systems were revealed be UV-vis measurements. Reflection spectroscopy and an integrating sphere were used in order to study the optical properties of the samples, in a Perkin Elmer Lambda 45 spectrophotometer (Waltham, MA, USA).

The magnetic measurements were performed with a superconducting quantum interference device (SQUID)—Quantum Design magnetometer (San Diego, CA, USA), in reciprocating sample oscillation (RSO) mode.

## 3. Results and Discussion

### 3.1. Equimolar Mixture β-Ga_2_O_3_ and α-Fe_2_O_3_ under HEBM 

#### 3.1.1. X-Ray Diffraction

**Figure 1:**
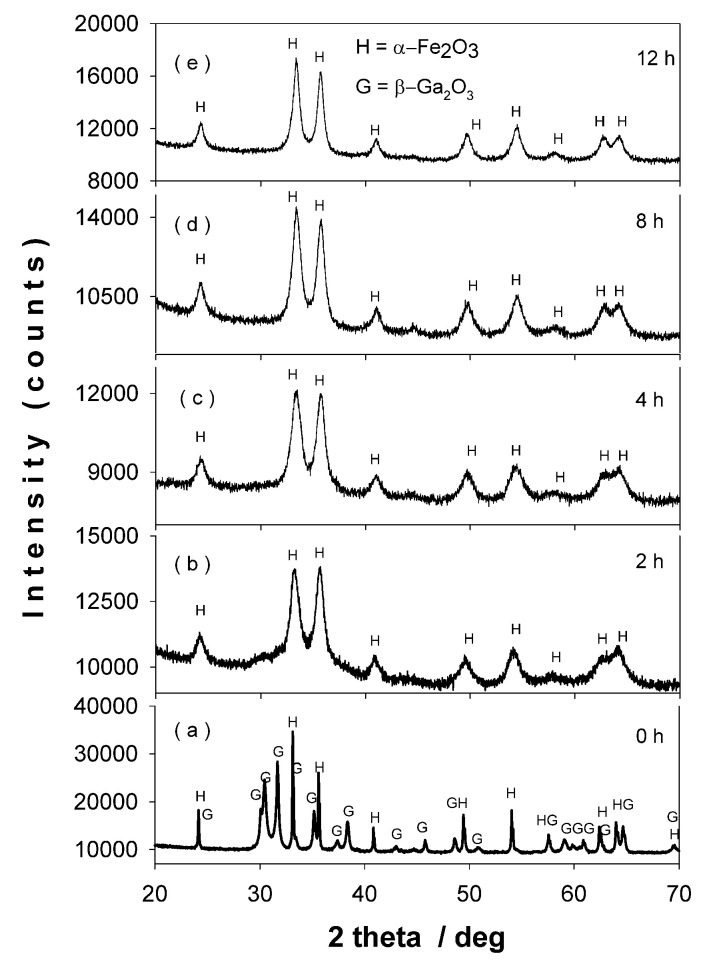
XRD patterns of the equimolar mixture β-Ga_2_O_3_–α-Fe_2_O_3_, corresponding to milling times: (**a**) 0 h, (**b**) 2 h, (**c**) 4 h, (**d**) 8 h and (**e**) 12 h.

**Figure 2:**
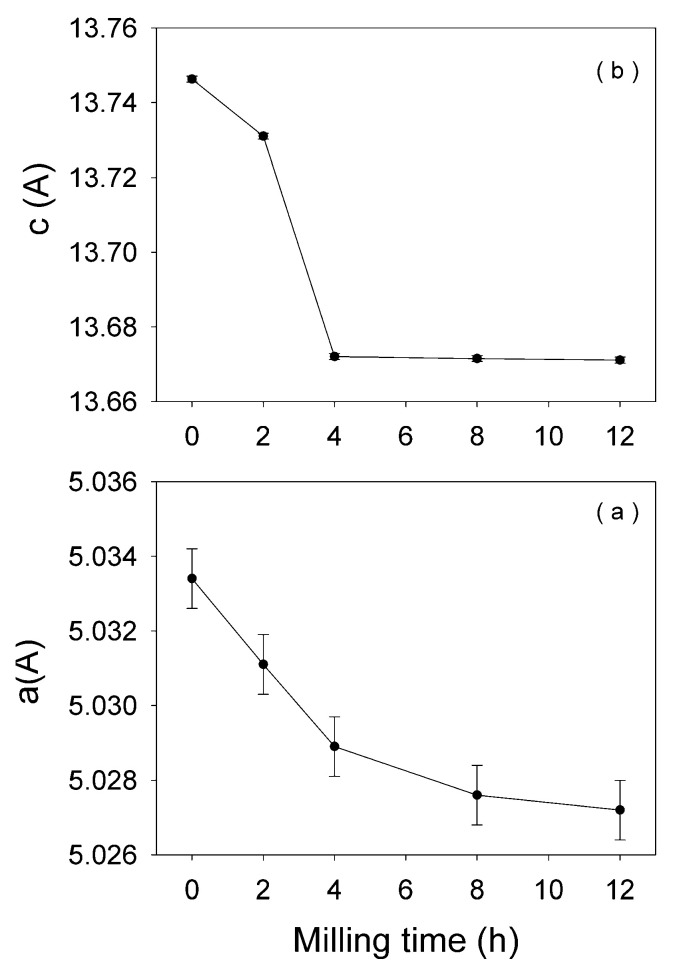
The lattice parameters c and a of hematite, as function of the ball milling time; (**a**) stand for a lattice parameter and (**b**) for c lattice parameter.

Figure 1a–e represents the X-ray diffraction (XRD) patterns of the equimolar mixture β-Ga_2_O_3_–α-Fe_2_O_3_, corresponding to milling times between 0 and 12 h. At 0 h of milling (Figure 1a), one can see the patterns of beta gallium and alpha iron oxides to be milled. In Figure 1b–e, XRD patterns reveal a progressive peak broadening with milling time, commonly ascribed to the decrease in crystallite size of the oxides under milling. After 2 h of ball milling the reflection lines of gallium oxide cannot be observed anymore. This behavior can be explained by two effects: a dissolution of Ga^3+^ ions into the hematite lattice, easily proven by the relevant intensity increase of the 110 reflection of hematite line and a drop of the c and a lattice constant (Figure 2), as well as by a small amorphization effect of gallium oxide due to HEBM process [25]. In Table 1, the lattice parameters, reliability R factors [26], crystallite size, and phase content given by the Rietveld refinement of XRD patters for β-Ga_2_O_3_—α-Fe_2_O_3_, subjected to HEBM from 2 h to12 h, are presented. A significant drop of crystallite size from ~100 to ~15 nm after 12 h of milling can be observed. The lattice parameters **c** and **a** of hematite (Figure 2 and Table 1) decreased as the ball milling time increased, indicating the dissolution of Ga^3+^ ions in the hematite lattice (Ga^3+^ ionic radius ~ 0.62 Å is smaller that of Fe^3+^ of about 0.67 Å). After 4 h or more of milling, the Ga- doped hematite is accompanied by a small amount (small percentage) of α-Fe coming from the ball collision process during high energy milling.

#### 3.1.2. ^57^Fe Mössbauer Spectroscopy

**Figure 3:**
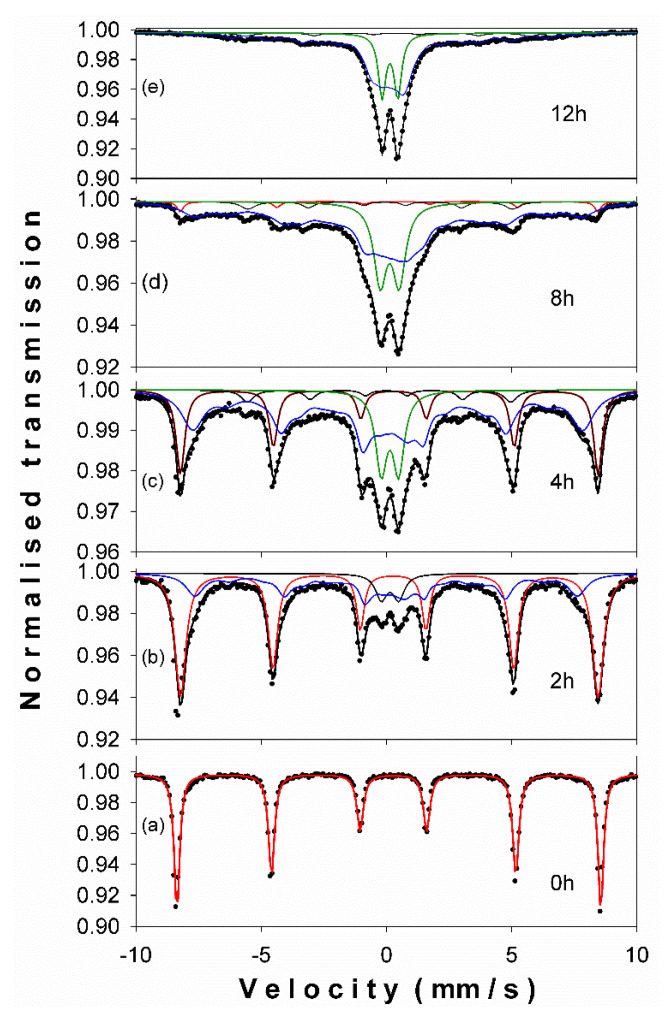
^57^Fe Mössbauer spectra recorded at room temperature for different ball milling times: (**a**) 0 h, (**b**) 2 h, (**c**) 4 h, (**d**) 8 h and (**e**) 12 h.

The room temperature Mössbauer spectra, corresponding to β-Ga_2_O_3_–α-Fe_2_O_3_ mixtures milled between 0 h and 12 h are displayed in Figure 3a–e. The characteristic Mössbauer hyperfine parameters (Isomer shift δ, Quadrupole splitting Δ, Hyperfine magnetic field B, at the ^57^Fe nucleus) phase assignment and relative abundance, given by the computer fit, are shown in Table 2. 

**Figure 4:**
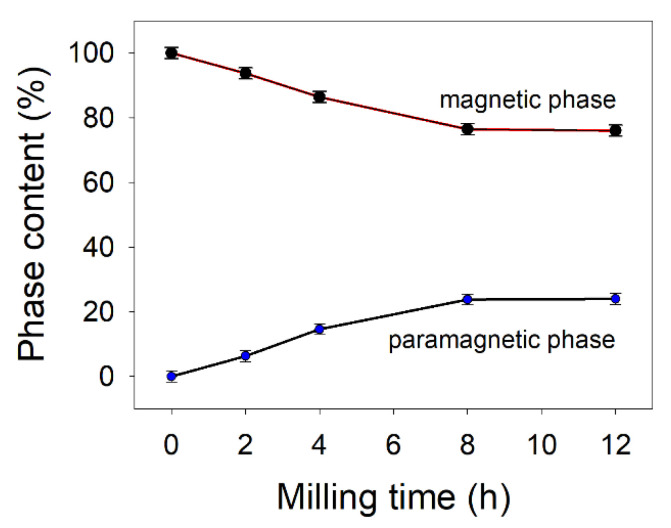
The evolution of paramagnetic to magnetic phases during the ball milling process.

**Figure 5:**
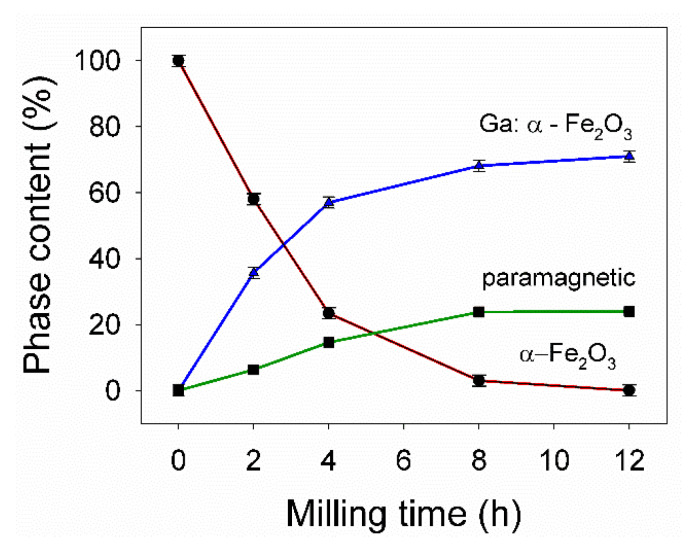
The phase evolution versus ball milling time.

At 0 h of milling, one can observe the typical six-line hematite pattern, with the characteristic negative quadrupole splitting (Δ) of −0.21 mm/s and a hyperfine magnetic field (B) of about 52 T. As the milling time increases, the Mössbauer spectra change. For 2 h of ball milling time, the Mössbauer spectrum was deconvoluted in a sextet corresponding to standard hematite, a hyperfine magnetic field distribution reflecting the disorder induced by the presence of gallium ions in the hematite lattice, and a central quadrupole doublet (paramagnetic phase, abundance of ~6%) coming from the contribution of nanoscaled hematite particles in the sample. After 4 h of milling (Figure 3c) the phase structure changes with a drop of pure hematite phase to ~23%, accompanied by an increase of the hyperfine field distribution component up to ~57% and of paramagnetic phase to ~14%. In good agreement with XRD data, one can observe the presence of α-Fe phase (~5%) represented by a characteristic sextet with B ≈ 33 T. For 8 h of ball milling time, the pure hematite phase dropped to ~3%, the hyperfine magnetic field distribution phase reached 68% from the spectrum area and the paramagnetic phase (central doublet) grew up to 24%, in agreement with the crystallite size drop in the system, as revealed by XRD refinements (Table 1). The magnetic gallium-doped hematite phase reached 71% after 12 h of milling (Figure 3e), while the paramagnetic phase was close to 24%, few percent belonging to the α-Fe phase. The evolution of the paramagnetic and magnetic phases percent, during the ball milling process of the equimolar mixture of β-Ga_2_O_3_–α-Fe_2_O_3_, is displayed in Figure 4. The increased paramagnetic phase, with ball milling time, is a consequence of crystallite size dropping. This phase is actually a gallium-doped hematite but with very small particle sizes (<7 nm). The phase evolution in the studied system is presented versus ball milling time in Figure 5. One can notice the quantitative decrease of the pure hematite phase and the progressive increase of the Ga-doped phase, as the milling time increases. Based on the above XRD and Mössbauer data, we can claim the facile synthesis of gallium-doped nanoscaled hematite, even at high molar contents of gallium. 

#### 3.1.3. TEM

**Figure 6:**
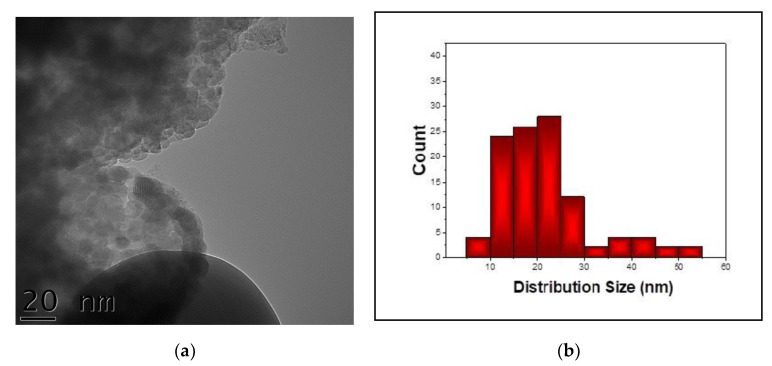
(**a**) TEM image on the gallium-doped hematite sample after 12 h of ball milling, and (**b**) the histogram for particle size distribution in the sample.

A general morphology image of the Ga-doped hematite sample after 12 h of ball milling time is given in Figure 6. The nano-particles’ structure can be noticed. Moreover, the mean particle size is of about ~20 nm, which is in agreement with the XRD results.

### 3.2. Obtaining and Characterizing the GaFeO_3_ (GFO)

The data presented in Section 3.1 demonstrate that in the case of equimolar oxide mixture, β-Ga_2_O_3_–α-Fe_2_O_3_, the energy generated by HEBM in the system was not enough to directly produce the crystallization of GaFeO_3_. Consequently, a series of calcinations up to 1000 °C was performed to find out the optimum temperature for the desired crystallization of GaFeO_3_.

#### 3.2.1. XRD Data

**Figure 7:**
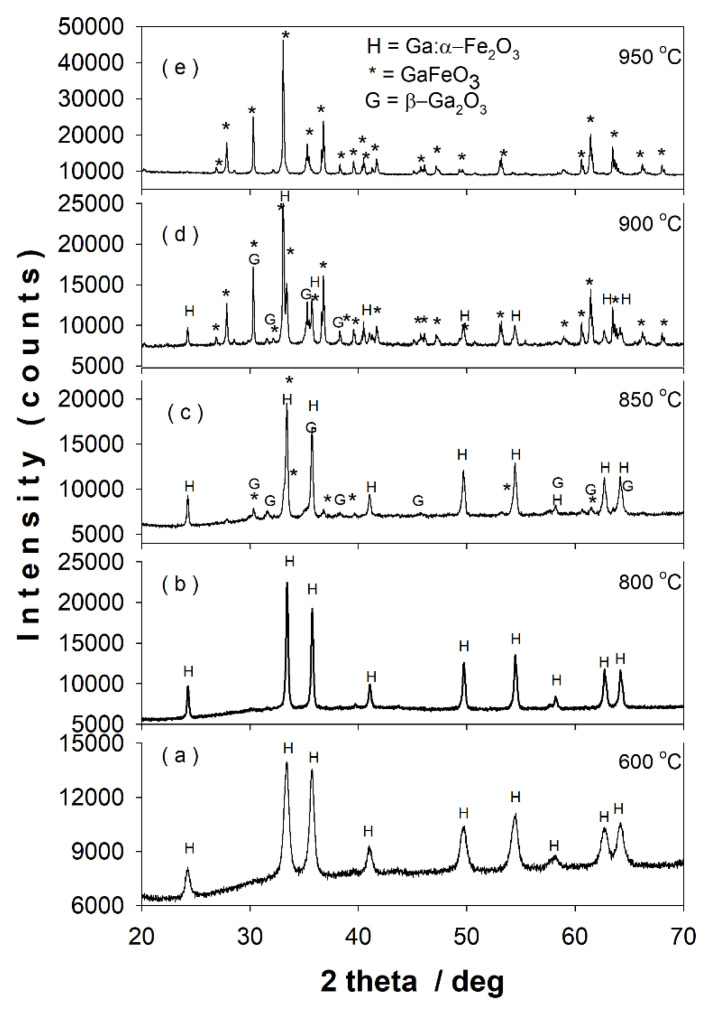
XRD patterns of the sample after 12 h of ball milling calcined at (**a**) 600 °C, (**b**) 800 °C, (**c**) 850 °C, (**d**) 900 °C, and (**e**) 950 °C.

Figure 7a–e shows the XRD patterns of the sample after 12 h of ball milling calcined at different temperatures in the range of 600–950 °C. 

Table 3 presents the lattice parameters, reliability R factors, crystallite sizes, and the phase content of the calcined samples as resulted from Rietveld structural refinements. At 600 °C and 800 °C (Figure 7a,b) only the nanoscaled gallium-doped hematite pattern can be observed. The crystallite size increases from ~21 nm at 600 °C to ~62 nm at 800 °C. The thermal treatment at 850 °C (Figure 7c) reveals the initiation of GaFeO_3_ formation (~13 wt. %) with the persistence of gallium-doped hematite (~75 wt. %). One can remark the unexpected appearance of β-Ga_2_O_3_ phase (~12 wt. %) in the XRD pattern of the sample calcined at 850 °C. The XRD pattern of the sample calcined at 900 °C (Figure 7d) consists in a prevailing GFO phase (~69 wt. %) together with gallium-doped hematite and small amounts of β-Ga_2_O_3_. At 950 °C (Figure 7e), the XRD pattern indicates only the presence of the single GaFeO_3_ phase. The calcination at 1000 °C leads also to the single phase of gallium orthoferrite with crystallite size greater than 100 nm.

The above XRD results show that, by first performing an energetic mechanical milling of the oxide mixture β-Ga_2_O_3_–α-Fe_2_O_3_ used to prepare the gallium ortho-ferrite, the calcination temperature drops by ~450 °C, in comparison with the solid-state reaction route [1].

#### 3.2.2. ^57^Fe Mössbauer Spectroscopy

**Figure 8:**
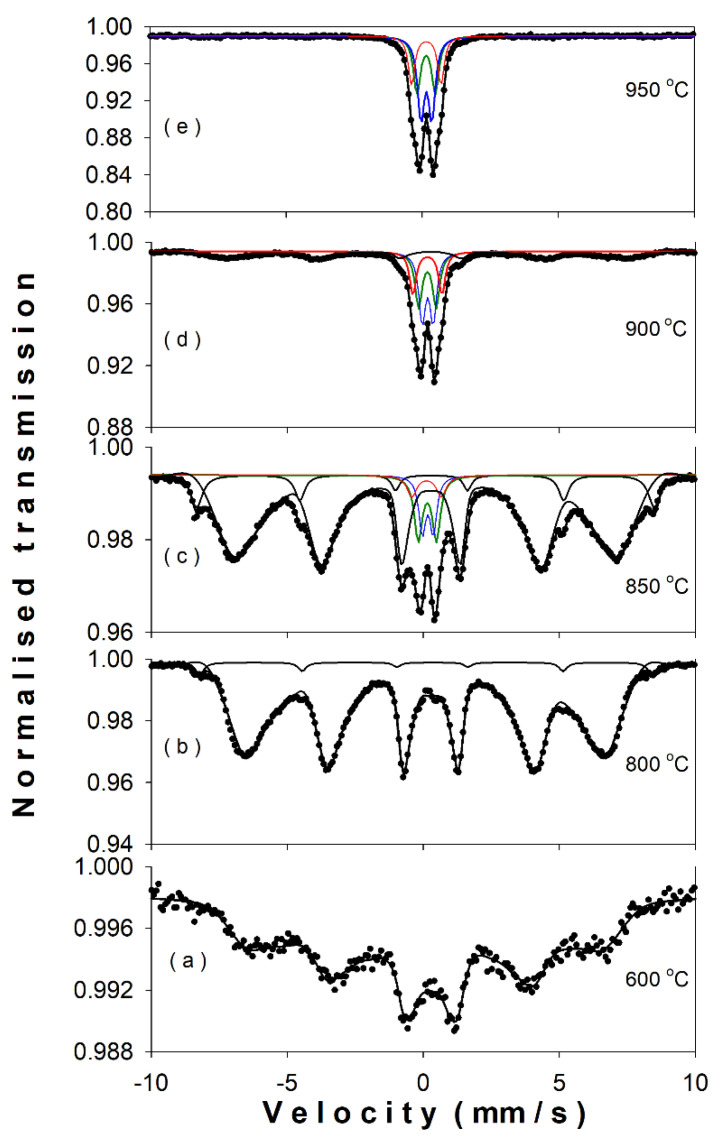
The Mössbauer spectra of the samples calcined at (**a**) 600 °C, (**b**) 800 °C, (**c**) 850 °C, (**d**) 900 °C, and (**e**) 950 °C.

The Mössbauer spectra of the samples calcined in the range of 600–1000 °C (Figure 8a–e) evolve from a magnetic pattern suggesting a distribution of hyperfine magnetic fields (Figure 8a) to a central quadrupole pattern characteristic to the gallium orthoferrite (Figure 8e). Table 4 presents the hyperfine parameters, site assignment, and phase abundance (as given by the computer fit with specialized programs). In Figure 8a, the spectrum of the sample at 600 °C was fitted best (continuous line) with a distribution of hyperfine magnetic fields reflecting the disorder made by the presence of gallium ions in the hematite lattice and probably by a large particle size distribution in the sample as well. At 800 °C, the Mössbauer pattern (Figure 8b) consists in a prevailing component of hyperfine magnetic field distribution accompanied by a small sextet typical for standard hematite. Figure 8c exhibits the spectrum at 850 °C indicating the presence of pure and gallium-doped hematite and the appearance of GaFeO_3_ phase (~15%), represented by the characteristic three quadrupole doublets (denoted S1, S2, and S3 in Table 4). If the spectrum at 900 °C (Figure 8d) is still revealing the presence of doped hematite (~30%), the spectrum at 950 °C (Figure 8e) indicates only the presence of pure GaFeO_3_. The three quadrupole doublets S1, S2, and S3 are ascribed to the nonequivalent Fe position in gallium orthoferrite structure [27]. One can claim that the formation of the GaFeO_3_ structure initiates in the temp range of ~800–850 °C, with previous ball milling of the samples.

**Figure 9:**
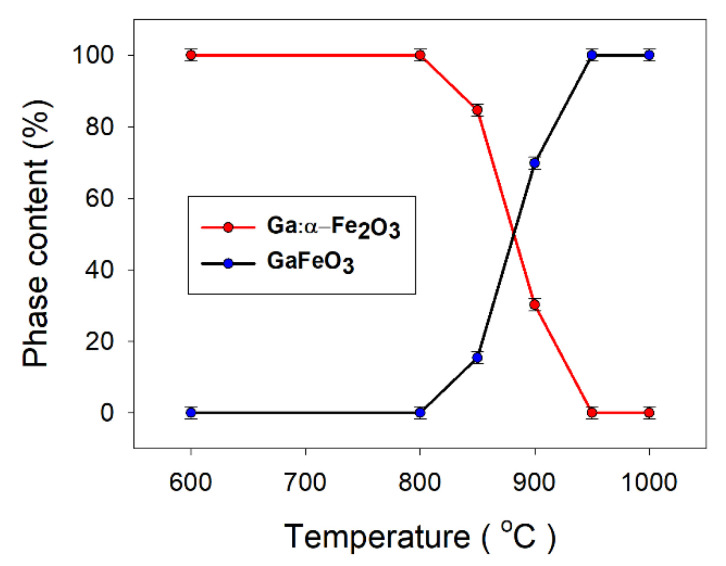
The phase evolution in the system versus treatment temperature, up to 1000 °C.

**Figure 10:**
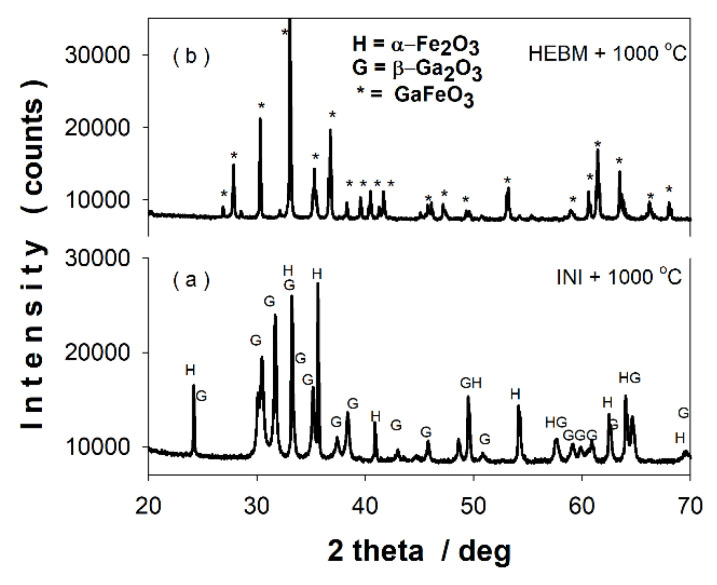
X-ray diffractograms of the initial mixture (**a**) and after milling (**b**), calcined at 1000 °C.

The phase evolution in the system versus treatment temperature (up to 1000 °C) is shown in Figure 9. Figure 10a,b emphasizes the key role of the energetic mechanical activation in significantly reducing the temperature required to obtain the gallium orthoferrite, compared with the common solid phase synthesis procedure. 

One notices in Figure 10a,b that the calcination at 1000 °C of the equimolar mixture β-Ga_2_O_3_–α-Fe_2_O_3_ was not enough to generate the crystallization of the desired gallium ortho-ferrite, while the same temperature (even 950 °C) was sufficient to obtain the pure phase GFO—starting with the same oxide mixture—that was first energetically milled for several hours.

#### 3.2.3. TEM Images

**Figure 11:**
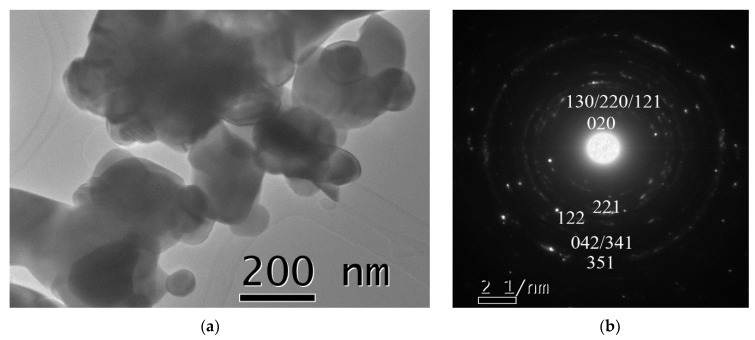
(**a**) The morphology of the GaFeO_3_ powder, obtained after 12 h of milling and 4 h of calcination at 950 °C of the equimolar mixture β-Ga_2_O_3_–α-Fe_2_O_3_; (**b**) selected area electron diffraction (SAED) pattern for the same sample.

Figure 11a,b displays the morphology and the selected area electron diffraction (SAED) pattern for the sample after 12 h of energetic milling followed by calcination for 4 h at 950 °C, proving the formation of the unique phase GaFeO_3_. The selected area electron diffraction (SAED) pattern shows a uniform distribution of diffraction spots in circles indicating the random crystallographic orientation of the GFO particles, further suggesting the polycrystalline nature of the material. The calcination process increased the particle size from ~20 nm to ~150 nm.

**Figure 12:**
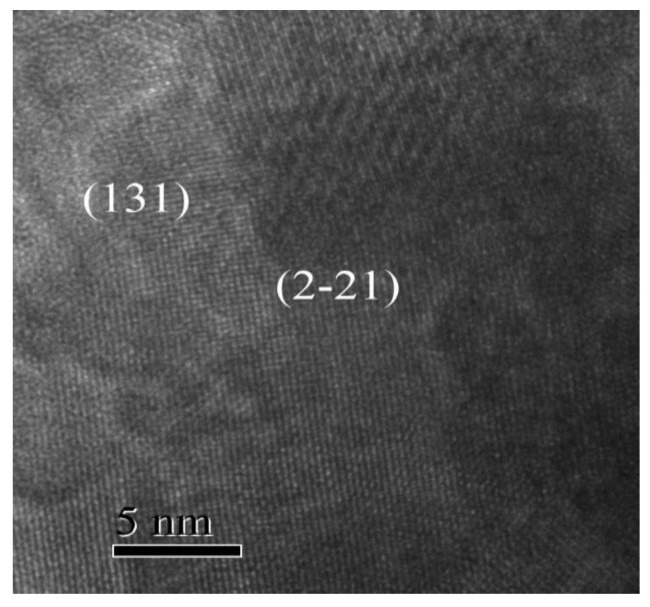
(HRTEM) image of GaFeO_3_ obtained at 950 °C.

High resolution transmission electron microscopy (HRTEM) image of the GFO sample is presented in Figure 12. The lattice fringes can be distinctly observed in Figure 12, showing the interplanar spacing corresponding to the (2 −2 1) and (1 3 1) planes of the GaFeO_3_ compound, spaced at 2.74 Å and 2.52 Å, respectively. The angle of 88 degrees between the two mentioned planes is close to the calculated theoretical value of 88.80 degrees.

### 3.3. Optical Properties of the Ga:α-Fe_2_O_3_ and GaFeO_3_ Systems

**Figure 13:**
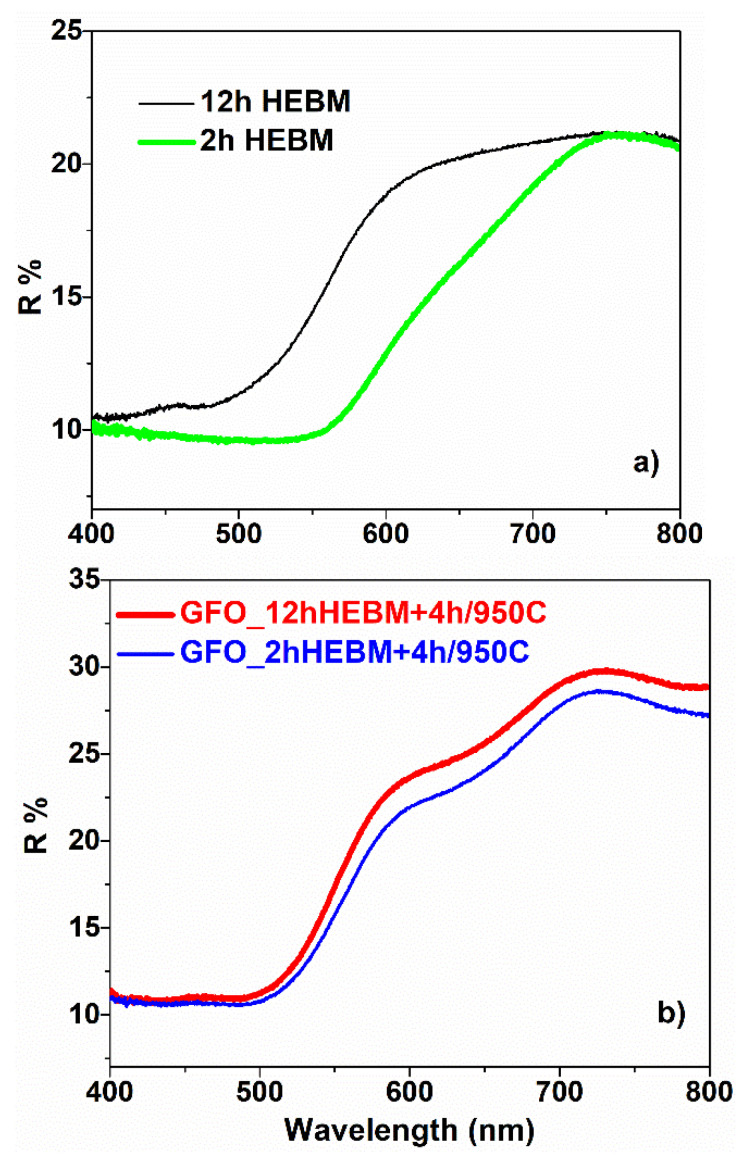
Reflection spectra of (**a**) Ga:α-Fe_2_O_3_ and (**b**) GaFeO_3_.

UV-vis measurements allowed us to obtain data on some optical properties of the investigated samples. The UV-vis absorption edge and band gap energy for all the samples were determined from the room temperature reflectance (R) spectra. The reflectance spectra of Ga:Fe_2_O_3_ and GaFeO_3_ are presented in Figure 13a,b. It can be observed that, for all the samples, the reflectance values present slopes with different angles at wavelengths between 500 nm and 600 nm.

In the case of Ga:α-Fe_2_O_3_, one can observe an increase of the reflectance (R) with milling time, for wavelengths higher than 500 nm, so the absorbance decreases in this range. A higher value of R was obtained for the sample subjected to HEBM for 12 h. The same behavior was evidenced in the case of GaFeO_3_; the lines in Figure 13b refer to the samples milled for 2 h and 12 h, respectively. 

**Figure 14:**
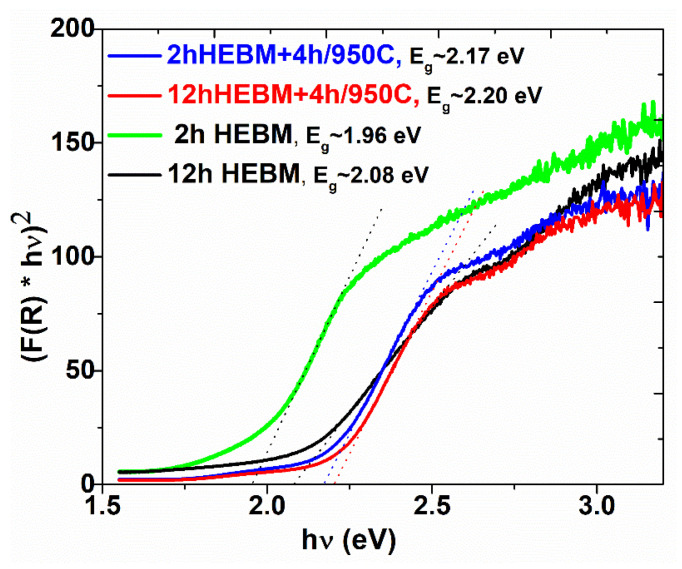
The Tauc graph from which the values of the band gap were extrapolated.

The band gap values of the samples were calculated using the representation of Tauc plot [28,29] as presented in Figure 14. Thus, using the Kubelka-Munk function F (R) = (1 − R)^1/2^/2R, where R is the diffuse reflectance, the Tauc’s plots (*F*(*R*) *hν*)*^n^* vs *hν*), where *hν* is the photon energy and *n* = ½ for direct band gap semiconductors, were represented for all samples. The band gap energies were estimated from the intersections of the tangents to the slopes in the Tauc’s plots with the photon energy axis. One notices that the higher the ball milling time, the bigger the band gap energy for both Ga:α-Fe_2_O_3_ and GaFeO_3_, expecting better properties toward visible spectrum region. The GFO shows values of Eg of about 2.2 eV, indicating possible photocatalytic properties at wavelengths higher than those corresponding to UV range.

### 3.4. Magnetic Properties of the Ga:α-Fe_2_O_3_ and GaFeO_3_ Systems

**Figure 15:**
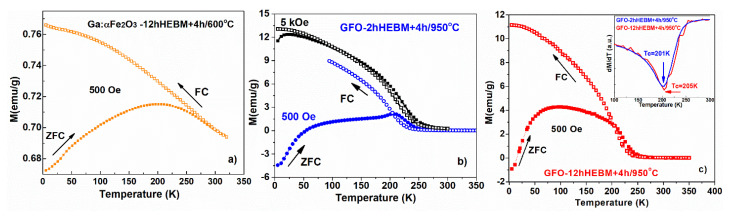
ZFC-FC termomagnetic measurements, recorded in 500 Oe applied magnetic field, for (**a**) Ga:Fe_2_O_3_; (**b**) gallium iron oxide (GFO) obtained after 2 h of milling and 4 h of calcination at 950 °C, with black squares representing ZFC-FC measured in 5 kOe; (**c**) GFO obtained after 12 h of milling and 4 h of calcination at 950 °C; inset: evaluation of the Curie temperature, from the dM/dT derivative.

Figure 15a shows the temperature dependence of the magnetization, measured according to the zero field cooled – field cooled (ZFC-FC) protocol, in a 500 Oe field, for the sample milled for 12 h and subsequently calcined 4 h at 600 °C (Ga:Fe_2_O_3_). One notices a superparamagnetic behavior of the Ga-doped hematite resulting from the divergence of the ZFC and FC curves below 300 K and the blocking temperature of 203 K. This behavior is consistent with the average of ~15 nm size of the crystallites, as resulted from XRD data analysis.

**Figure 16:**
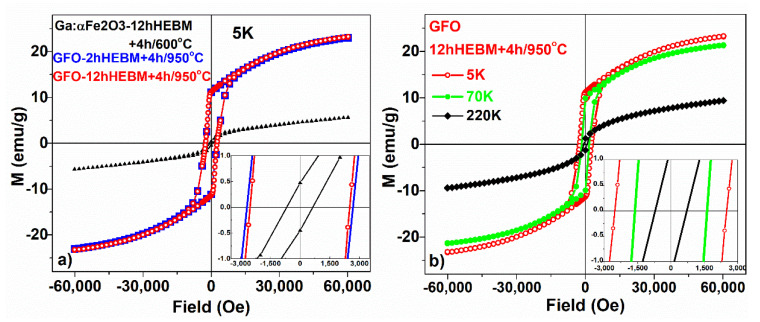
Hysteresis curves measured (**a**) at 5 K for Ga:Fe_2_O_3_ and for GaFeO_3_ obtained after previous milling for 2 h and 4 h of calcination at 950 °C (full blue symbols) and 12 h of milling and calcination (open symbols), respectively; inset: zoomed view to determine coercitivity; (**b**) for GaFeO_3_ obtained after 12 h of milling and calcination 4 h/950 °C at 5 K, 70 K, and 220 K; inset: zoomed view to determine coercitivity.

The samples calcinated at higher temperatures, in which the gallium orthoferrite (GFO) was formed, showed a totally different behavior of the magnetization as function of temperature. The calcined samples (for 4 h at 950 °C), in which GFO was obtained, showed a similar temperature dependence of magnetization, regardless of the initial milling time (e.g., the samples milled for 2 h and 12 h, respectively)—see Figure 15b,c. Similar behavior was also found in the magnetization isotherms, measured at 5 K, none of the measured samples reaching saturation, even for an applied field of 6T (see Figure 16a), which suggests a high anisotropy of the compound [30]. The GFO sample obtained after 2 h of milling time, followed by a calcination of 4 h/950 °C, had a slightly higher coercitive field, Hc = 2690 Oe, compared to the one obtained after milling for 12 h, which had Hc = 2533 Oe. For the Ga:Fe_2_O_3_ compound, we obtained Hc = 670 Oe. 

To have an insight into the temperature dependence of magnetization, the samples were first cooled in zero field (ZFC) and then a 500 Oe was applied on heating. One notices that at low temperatures the magnetization shows negative values for both calcinated samples (Figure 15b,c). First, we must mention that this negative magnetization is not an artifact due to the remnant magnetic field of the SQUID, which was measured to be less than 10 Oe, compared to the applied field of 500 Oe—for measuring the ZFC curves. 

The phenomenon of negative magnetization (not due to diamagnetism and accompanied with a magnetization reversal with increasing temperature) is known in literature and has been associated with intrinsic parameters such as: crystal structure, magnetic anisotropy, magnetic exchange interactions, and temperature dependence of sublattice magnetization [31]. The magnetic anisotropy is a key property preventing the rotation of the net magnetic moments in the direction of the applied field, below the compensation temperature.

The study reported by [15] highlights a strong magnetic anisotropy of single crystal GFO. The crystalline structure of GFO, orthorhombic with a space group of Pna21, with four Fe sites, two distorted and one regular octahedral and another one tetrahedral, leads to a complex magnetic behavior of these compounds. In our samples, a strong magnetic anisotropy is suggested by the larger c axis determined from the XRD analysis (around 9.3973 Å), the high coercivity at 5 K, and the fact that even for an applied field of 6T none of the measured samples reach saturation (see Figure 16a) [30]. The strong anisotropy of our samples is a prerequisite for the occurrence of negative magnetization.

Another essential condition for the appearance of the negative magnetization phenomenon is the antiparallel ordering between two or more magnetic components showing different temperature dependences of their magnetizations below their magnetic ordering temperatures under the influence of strong magnetic anisotropies [31]. In this context, we have seen studies showing that, in the GFO, the Ga atoms can occupy Fe sites resulting in the formation of “two sublattices constituted by the Ga and Fe sites” [16] with different values of the magnetic moments and probably different temperature dependences, leading to ferrimagnetism. It was shown both theoretically and experimentally that structural disorder may induce changes in magnetic behaviour [30,32,33], for example the strength of the exchange interaction between Fe^3+^ ions can be modified by the Ga atoms occupying Fe sites [30]. Moreover, a net magnetization of the GFO can arise from uncompensated ordering of neighbouring sub-lattices [12]. 

The GFO obtained after 2 h of milling and 4 h of calcination at 950 °C shows a maximum (i.e., a cusp in the temperature dependence of magnetization) before reaching the magnetic order transition. The magnetic order-disorder transition temperatures were determined as the differential minimum from the first derivative of the temperature dependent magnetization on the field cooling (FC) (see inset of Figure 15c). For the GFO sample obtained after 2 h of milling and 4 h of calcination at 950 °C the Curie ferromagnetic–paramagnetic transition (Tc) takes place at 201 K, while for the GFO obtained after 12 h of milling and 4 h of calcination at 950 °C, we obtained 205 K as transition temperature. The obtained values for the magnetic order-disorder transitions are comparable with the values obtained on GFO prepared by solid state reaction in [1,12].

On cooling the samples in 500 Oe, i.e., the FC curves in Figure 15, a divergence was noticed compared to the ZFC curve. The difference between FC and ZFC magnetizations is known to be related to the magnitude and the temperature variation of coercivity, which is a measure of the magnetic anisotropy [34]. A large coercitivity at low temperatures leads to the increase of the FC magnetization.

The hysteresis curves measured for GaFeO_3_ obtained after 12 h of HEBM and calcination for 4 h/950 °C at 5 K, 70 K, and 220K are shown in Figure 16b. The coercitive field is much higher at 5 K (2533 Oe), compared to 70 K (1618 Oe) and 220 K (719 Oe). As mentioned in [12], since coercivity is related to magnetic anisotropy, the irreversible magnetic behavior reflects the role of anisotropy in determining the shapes of FC and ZFC curves below the ordering temperature.

The cusp noticed for the GaFeO_3_ sample obtained after 2 h milling and calcinated 4 h at 950 °C moves to much lower temperatures if the applied field increases from 500 Oe to 5000 Oe (see Figure 15b, black curve, the cusp moving at approx. 25 K). The bifurcation between the ZFC and FC samples and the presence as well as the temperature behavior of the mentioned cusp, originate in the anisotropy field of the GFO [12,30,32]. The origin of magneto-crystalline anisotropy in GFO received a possible explanation in terms of the large orbital angular momentum due to the off-center displacement of Fe^3+^ ions [30].

## 4. Conclusions

GaFeO_3_ (GFO) ortho-ferrite was synthesized by high-energy ball milling (HEBM) and post-annealing pathway starting with an equimolar mixture of β-Ga_2_O_3_ and α-Fe_2_O_3_. X-ray powder diffraction, ^57^Fe Mössbauer spectroscopy, and transmission electron microscopy were used to study the phase evolution in β-Ga_2_O_3_–α-Fe_2_O_3_ system under HEBM conditions, as well as after calcination. Energetic mechanical milling for 2–12 h and subsequent annealing up to 1000 °C were performed. Pure, well-crystallized GFO phase was obtained after 12 h of milling and post-annealing pathway at 950 °C (4 h). This reduced annealing temperatures and times are the main advantages of our preparation route. Commonly the GFO is prepared by solid phase reaction between β-Ga_2_O_3_ and α-Fe_2_O_3_ at relative high temperature (~1400 °C) and long reaction time (5–20 h). Our results evidence that both mechanical milling and calcination are important; it was shown that the calcination of the initial β-Ga_2_O_3_–α-Fe_2_O_3_ mixture up to 1000 °C results in a gallium-doped hematite-like phase. The GFO structure was obtained only after calcination at 950 °C of the oxide mixture β-Ga_2_O_3_–α-Fe_2_O_3_, previously subjected to HEBM for 12 hours. The magnetic and optic properties of the investigated samples were revealed by magnetic and UV-vis measurements respectively. The Ga-doped hematite exhibits superparamagnetic behavior with a blocking temperature of 203 K. It was found that the magnetic properties of the final product GaFeO_3_ were independent of the milling time of the initial oxide mixture β-Ga_2_O_3_–α-Fe_2_O_3_, in the range of 2–12 h. The band gap energy (E_g_) determined from Tauc’s plots was close to 2.2 eV, suggesting possible applications as photocatalytic material. This work also evidences the crucial role of energetic mechanical activation in the synthesis of GaFeO_3_ ortho-ferrite via mechanochemistry.

## Figures and Tables

**Table 1:** Lattice parameters, reliability R factors, crystallite size, and phase content in the Rietveld refinement of XRD patters for β-Ga_2_O_3_–α-Fe_2_O_3_ (HEBM@0–12 h) system.

Milling Time (h)	Lattice Parameters (Å)			Reliability R Factors (%)			Crystallite Size (nm)	Phase Content (wt. %)
	*a*	*b*	*c*	R_p_	R_wp_	R_exp_		
0	5.0334 12.2143	- 3.0384	13.7463 5.8084	1.05	1.43	3.69	>100 >100	α-Fe_2_O_3_ (21.3) β-Ga_2_O_3_ (78.7)
2	5.0311	-	13.7310	1.26	1.68	7.23	14.1	Ga:α-Fe_2_O_3_ (100)
4	5.0289 2.8640	- -	13.672 -	1.15	1.51	7.44	12.2 95.2	Ga:α-Fe_2_O_3_ (98.1) α- Fe (1.9)
8	5.0276 2.8690	- -	13.6715 -	1.29	1.77	8.14	13.9 93.6	Ga:α-Fe_2_O_3_ (97.3) α- Fe (2.7)
12	5.0272 2.8589	- -	13.6711 -	1.05	1.43	8.94	15.6 94.6	Ga:α-Fe_2_O_3_ (97.8) α- Fe (2.2)
Errors	±0.0005	-	±0.0005				±1.5	±1.2

**Table 2:** Mössbauer hyperfine parameters for system β-Ga_2_O_3_—α-Fe_2_O_3_, after HEBM.

Milling Time (h)	δ * (mm/s)	Δ (mm/s)	B (T)	Phase Assignment	Relative Abundance (%)
0	0.288	−0.209	52.4	α-Fe_2_O_3_	100
2	0.293 0.258 0.254	−0.178 −0.350 0.705	51.7 4.6–47.4 -	α-Fe_2_O_3_ Ga:α-Fe_2_O_3_ Paramagnetic	58.0 35.7 6.3
4	0.295 0.262 0.268 0.254	−0.197 −0.231 −0.300 0.699	51.8 5.7–48.3 32.7 -	α-Fe_2_O_3_ Ga:α-Fe_2_O_3_ α-Fe Paramagnetic	23.4 57.0 5.0 14.6
8	0.360 0.293 0.204 0.244	−0.341 −0.243 −0.197 0.760	51.9 5.5–47.9 32.9 -	α-Fe_2_O_3_ Ga:α-Fe_2_O_3_ α-Fe Paramagnetic	3.0 68.1 5.1 23.8
12	0.159 0.334 0.252	−0.090 −0.362 0.620	4.3–47.9 33.2 -	Ga:α-Fe_2_O_3_ α-Fe Paramagnetic	71.0 5.0 24.0
Errors	±0.005	±0.010	±0.5		±0.06

* The isomer shift δ is given to α-Fe.

**Table 3:** Lattice parameters, reliability R factors, crystallite size, and phase content in the Rietveld refinement of XRD patters for β-Ga_2_O_3_–α-Fe_2_O_3_ (HEBM@0–12 h) system, followed by calcination between 600–1000 °C.

Temperature (°C)	Lattice Parameters (Å)			Reliability R Factors (%)			Crystallite Size (nm)	Phase Content (wt. %)
	*a*	*b*	*c*	R_p_	R_wp_	R_exp_		
600	5.0223	-	13.6720	1.38	1.85	7.62	20.9	Ga:α-Fe_2_O_3_ (100)
800	5.0261	-	13.6720	2.15	3.75	12.75	62.2	Ga:α-Fe_2_O_3_ (100)
850	5.0249 12.3140 5.0932	- 3.0382 8.7406	13.6720 5.8413 9.3867	2.37	3.89	16.90	65.1 13.9 40.7	Ga:α-Fe_2_O_3_ (74.52) β-Ga_2_O_3_ (12.46) GaFeO_3_ (13.02)
900	5.0312 12.3140 5.0856	- 3.0234 8.7527	13.6720 5.8553 9.4123	1.70	2.74	18.56	54.6 - 68.4	Ga:α-Fe_2_O_3_ (27.79) β-Ga_2_O_3_ (3.52) GaFeO_3_ (68.69)
950	5.0839	8.7498	9.3973	2.03	3.25	22.46	>>100	GaFeO_3_ (100)
1000	5.0829	8.7476	9.3963	1.99	3.22	24.71	>>100	GaFeO_3_ (100)
Errors	±0.0005	±0.0005	±0.0005				±1.5	±1.2

**Table 4:** Mössbauer hyperfine parameters for the system β-Ga_2_O_3_–α-Fe_2_O_3_ after 12 h of milling and calcination at different temperatures.

Temperature (°C)	δ * (mm/s)	Δ (mm/s)	B (T)	Phase Areas (%)	Phase Assignment
600	0.280	−0.23	6.9–49.2	100	Ga:α-Fe_2_O_3_
800	0.277 0.310	−0.232 −0.268	5.2–49.8 51.5	97.0 3.0	Ga:α-Fe_2_O_3_ α-Fe_2_O_3_
850	0.284 0.175 0.259 0.277 0.229	−0.216 −0.247 0.671 0.410 1.091	7.4–52.6 52.1 - - -	75.5 10 6.7 5.0 2.8	Ga:α-Fe_2_O_3_ α-Fe_2_O_3_ S1-GaFeO_3_ S2-GaFeO_3_ S3-GaFeO_3_
900	0.295 0.262 0.272 0.263	−0.169 0.648 0.409 1.075	16.4–46.3 - - -	30 24 28 18	Ga:α-Fe_2_O_3_ S1-GaFeO_3_ S2-GaFeO_3_ S3-GaFeO_3_
950	0.260 0.269 0.263	0.677 0.406 1.086	- - -	32 43 25	S1-GaFeO_3_ S2-GaFeO_3_ S3-GaFeO_3_
1000	0.260 0.269 0.266	0.681 0.405 1.086	- - -	32 43 25	S1-GaFeO_3_ S2-GaFeO_3_ S3-GaFeO_3_
Errors	±0.005	±0.010	±0.5	±0.06	

* The isomer shift δ is given relative to α-iron.

## Data Availability

Data can be provided by the corresponding author upon reasonable request.
